# Herbal Composition Inhibits Mitochondrial Oxidative Phosphorylation to Prevent HER2-Positive Breast Cancer and Identifies Potential Active Compounds

**DOI:** 10.3390/ijms262411970

**Published:** 2025-12-12

**Authors:** Yi Zhao, Wenxiu Hu, Xinnan Wang, Zhiyue Ren, Yumeng Gong, Lu Liu, Youzhi Sun

**Affiliations:** 1 Research Center for Differentiation and Development of Basic Theory of Traditional Chinese Medicine, Jiangxi University of Chinese Medicine, Nanchang 330004, China; hwxhbhg@outlook.com (W.H.); 13065138090@163.com (X.W.); 17335578099@163.com (Z.R.); sanyer0496@163.com (Y.G.); ll017620@163.com (L.L.); 2 School of Chinese Medicine, Jiangxi University of Chinese Medicine, Nanchang 330004, China

**Keywords:** three herbal compositions SLC, active components, PDK1/PDHA1 signaling pathway, SIRT1/PGC-1α/NRF1/TFAM signaling pathways, molecular docking, HER2-positive breast cancer

## Abstract

Human Epidermal Growth Factor Receptor 2 (HER2)-positive breast cancer is an aggressive malignancy with limited treatment options. The herbal composition SLC contains *Salvia miltiorrhiza* Bunge (Dan shen), *Ligusticum wallichii* Franch. (Chuan xiong), and *Carthamus tinctorius* L. (Hong hua), three herbs that have demonstrated antitumor properties. This study aims to investigate the inhibitory effects and mechanisms of SLC against HER2-positive breast cancer. UPLC-Q/TOF-MS identified 113 compounds in SLC. SLC inhibited the proliferation, migration, and mitochondrial function of HER2-positive cells by reducing glucose uptake, ATP production, and oxygen consumption rate (OCR). Furthermore, SLC downregulated the levels of p-HER2/HER2, p-AKT/AKT, and p-ERK/ERK by Western blot, thereby inhibiting the HER2 signaling pathway. Mechanistically, SLC decreased the protein expression of PDK1 and inhibited the phosphorylation of PDHA1 (Ser293), and also inhibited mitochondrial-related proteins in SIRT1/PGC-1α/NRF1/TFAM signaling axes. Additionally, through the overexpression of PDK1, SLC repressed PDK1, downregulated PDHA1, and induced apoptosis. In vivo xenograft model studies demonstrated that SLC inhibited tumor growth. Molecular docking highlighted Monomethyl lithospermate as a key active component. Overall, SLC influences oxidative phosphorylation via the PDK1/PDHA1 and SIRT1/PGC-1α/NRF1/TFAM signaling pathways and downregulates the HER2 pathway, thereby ultimately inhibiting HER2-positive breast cancer progression.

## 1. Introduction

HER2-positive breast cancer is a highly aggressive subtype that represents 15% to 20% of all breast cancer cases [1]. HER2 is a transmembrane tyrosine kinase receptor that plays a role in cell growth, differentiation, and survival [2]. Recent research indicates that HER2-positive breast cancer consists of a diverse group of diseases, with HER2 overexpression leading to the loss of cell–cell junctions, making it more aggressive than luminal breast cancer cells [2,3,4]. Trastuzumab is the primary treatment option for HER2-positive breast cancer [5,6]. However, resistance to trastuzumab is prevalent, posing a significant challenge in managing HER2-positive breast cancer, with up to 23% of patients experiencing a recurrence within ten years [7,8]. Therefore, it is essential to investigate new therapeutic targets or strategies for treating HER2-positive breast cancer.

Metabolic reprogramming is widely acknowledged as a hallmark of breast cancer, playing a critical role in disease progression by promoting uncontrolled cellular proliferation, metastasis, and resistance to therapy, thereby constituting a promising target for therapeutic intervention [9,10]. While the majority of cancer cells utilize both aerobic glycolysis and mitochondrial oxidative phosphorylation (OXPHOS) to generate energy, overexpression of HER2 has been demonstrated to augment glycolysis, manifested by increased glucose uptake and lactate production—concomitant with a reduction in oxygen consumption in breast cancer models [11]. Furthermore, metabolic processes are highly dynamic and are modulated by interactions between tumor cells and their microenvironment [12]. Among the various microenvironmental factors, hypoxia represents a particularly potent driver of the metabolic shift from OXPHOS toward glycolysis [13]. This metabolic plasticity facilitates the rewiring of mitochondrial pathways, thereby influencing breast cancer progression and enabling mitochondria to transmit signals that regulate nuclear oncogenic programs [14]. Accordingly, OXPHOS has emerged as a potential target for cancer therapy [15]. The role and regulation of metabolic reprogramming in HER2-positive breast cancer, especially under hypoxic conditions, remain inadequately understood. This gap in knowledge is critical and carries significant implications for therapeutic development.

Current research indicates that the pyruvate dehydrogenase complex (PDC), with particular emphasis on its critical pyruvate dehydrogenase E1 alpha 1 subunit (PDHA1), is integral to mitochondrial oxidative phosphorylation (OXPHOS) and the tricarboxylic acid cycle [16]. The activity of PDHA1 is negatively regulated by pyruvate dehydrogenase kinase 1 (PDK1), which phosphorylates PDHA1 at the Ser232 residue, thereby facilitating a metabolic shift toward glycolysis—a phenomenon frequently observed in tumor cells [17]. PDK1 has been implicated in various malignant processes, including cellular proliferation, evasion of apoptosis, metastasis, and poor clinical prognosis [18]. Furthermore, PDK1 is a direct transcriptional target of hypoxia-inducible factor 1-alpha (HIF-1α), establishing a mechanistic link between hypoxic conditions and glycolytic reprogramming in breast cancer [19,20]. From a pharmacological perspective, the PDK1 inhibitor dichloroacetate (DCA) has demonstrated the capacity to reverse this metabolic shift by enhancing PDH activity and restoring mitochondrial respiration [21]. Collectively, these findings highlight the critical role of the PDC/PDK/PDHA1 axis in metabolic reprogramming and underscore its potential as a therapeutic target in oncology [22].

Conversely, proliferator-activated receptor gamma coactivator 1-alpha (PGC-1α) functions as a principal regulator of mitochondrial OXPHOS. Inhibition of PGC-1α has been shown to suppress OXPHOS, impede cellular proliferation, and inhibit tumor growth [23]. The deacetylation of PGC-1α by Silent mating type information regulation 2 homolog 1 (SIRT1) activates downstream effectors, including nuclear respiratory factor 1 (NRF1) and mitochondrial transcription factor A (TFAM), which collectively promote mitochondrial biogenesis and enhance respiratory chain function [24]. Notably, emerging evidence suggests that OXPHOS is upregulated in certain malignancies and contributes to tumor progression, thereby positioning it as a promising therapeutic target [25].

Traditional Chinese medicine plays an important role in treating tumors by regulating energy metabolism [26]. In our investigation, we utilized an herbal formulation comprising three specific herbs: *Salvia miltiorrhiza* Bunge (Dan shen), *Ligusticum wallichii* Franch. (Chuan xiong), and *Carthamus tinctorius* L. (Hong hua), collectively referred to as SLC, which was modified from Guanxin II [27]. Guanxin II, developed by Chen Keji, a National Master of Traditional Chinese Medicine, exhibits neuroprotective properties. Notably, its simplified formulation, Danshen-Chuanxiong-Honghua (DCH), has been demonstrated clinically to alleviate cerebral impairment and enhance spatial cognitive function [28]. Contemporary pharmacological research has substantiated the presence of numerous bioactive compounds within these herbs that exhibit anti-tumor properties. For instance, *Salvia miltiorrhiza* Bunge and its active constituents have been demonstrated to inhibit cellular proliferation, metastasis, and angiogenesis, while also inducing apoptosis and autophagy, and modulating immune responses and the tumor microenvironment [29]. Additionally, extracts from *Carthamus tinctorius* L. have been shown to suppress the expression of metastatic genes in MDA-MB-231 breast cancer cells [30]. Furthermore, alcohol extracts of Ligusticum chuanxiong Hort. have been reported to possess anticancer effects and to mitigate multidrug resistance in cancer cells [31]. These findings suggest that the SLC herbal recipe may hold promise as a potential therapeutic agent against breast cancer.

Consequently, the objective of this study was to investigate whether SLC inhibits the HER2 signaling pathway, suppresses the PDK1/PDHA1 signaling pathway, and modulates the SIRT1/PGC-1α/NRF1/TFAM signaling axis, ultimately leading to the inhibition of oxidative phosphorylation (OXPHOS) in HER2-positive breast cancer cells. Finally, through molecular docking technology, the active components targeting PDK1 were discovered and confirmed.

## 2. Results

### 2.1. Component Analysis of SLC by UHPLC-Q-TOF/MS

**Figure 1:**
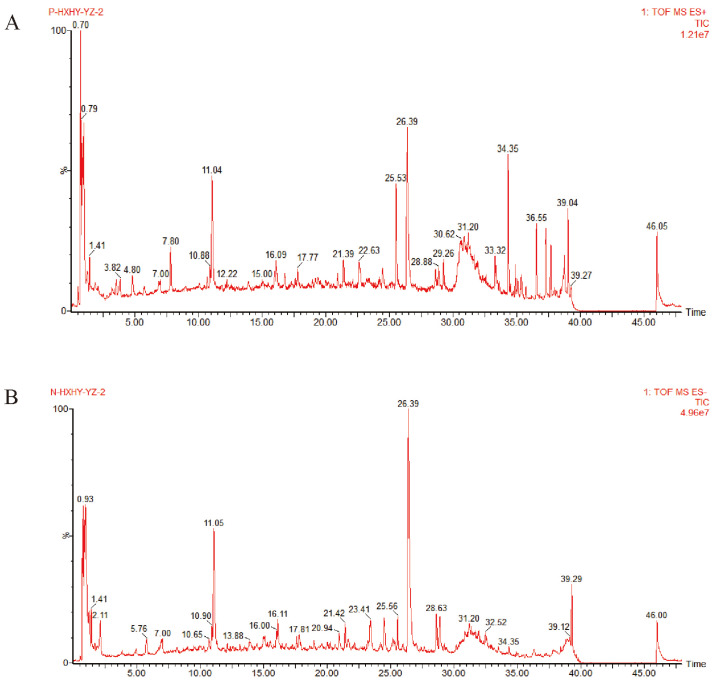
The Peak ion chromatogram (TIC) of SLC in positive and negative ion mode. (**A**). Positive ion mode; (**B**). Negative ion mode.

The total ion chromatograms of SLC provided insights into the chemical composition of the constituent compounds (Figure 1). A comprehensive search of the PubChem, Scifinder, and Chemicalbook databases yielded information on the chemical compositions of three herbs, resulting in a compilation of 714 compounds. This data was subsequently imported into UNIFI 1.9 software, leading to the identification of 113 active ingredients, including chuanxiongnolide L1, naringin, hydroxysafflor yellow B, methyltanshinonate, and tetramethylpyrazine. Specifically, 36 ingredients were derived from *Salvia miltiorrhiza* Bunge, 36 from *Ligusticum wallichii* Franch, and 42 from *Carthamus tinctorius* L. The identified compounds included 20 acids, 20 flavonoids, 15 esters, 9 lactones, 8 ketones, 6 alcohols, 3 pigments, 3 alkaloids, 3 glycosides, 2 amino acids, and 25 additional compounds. A detailed inventory of these ingredients is provided in Supplementary File S1, Table S1.

### 2.2. Colony Formation, Wound Healing, and Cell Migration Assays

**Figure 2:**
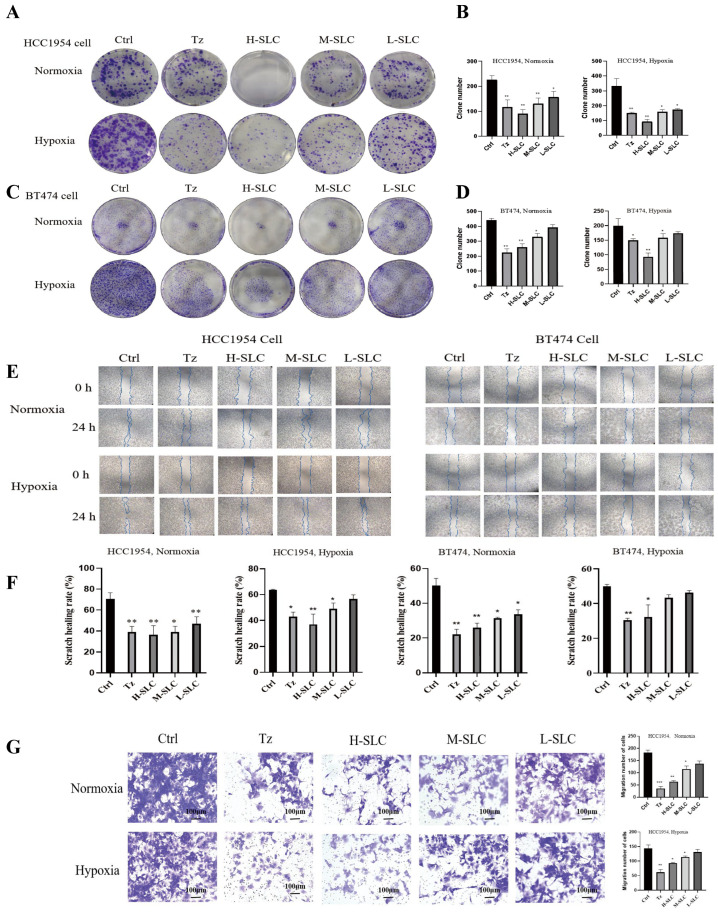
SLC effect on HER2-positive breast cancer cell proliferation, wounding heals and migration under normoxic and hypoxic conditions. (**A**,**B**): Colony formation after SLC intervention on HCC1954 cells. (**C**,**D**): Colony formation after SLC intervention on BT474 cells. (**E**,**F**): Wound healing assay after SLC intervention on HCC1954 and BT474 cells. (**G**) The migration ability of SLC was evaluated using a transwell assay on HCC1954 cells. N = 3. Tz is the abbreviation for Trastuzumab, with a dosage of 25 μg/mL; H-SLC, M-SLC, and L-SLC represent high, medium, and low doses of SLC, which are 2500 μg/mL, 1250 μg/mL, and 625 μg/mL, respectively. Compared with Ctrl group, * *p* < 0.05, ** *p* < 0.01, *** *p* < 0.001.

The anti-proliferative effects of SLC were assessed through a colony formation assay. The results, illustrated in Figure 2A,B, indicate that Tz, H-SLC, M-SLC and L-SLC all significantly suppressed the proliferation of HCC1954 cells under both normoxic and hypoxic conditions when compared to the Ctrl group (*p* < 0.001, *p* < 0.01, or *p* < 0.05). In the case of BT474 cells, as depicted in Figure 2C,D, only the Tz, H-SLC, and M-SLC groups showed inhibitory effects under both normoxic and hypoxic conditions (*p* < 0.01 or *p* < 0.05). In wound healing experiments, as demonstrated in Figure 2E,F, under normoxic conditions, H-SLC, M-SLC, and L-SLC all exhibit inhibitory effects on BT474 and HCC1954 cells. However, under hypoxic conditions, the inhibitory effects of SLC are attenuated. Specifically, in HCC1954 cells, only H-SLC and M-SLC demonstrate inhibitory activity (*p* < 0.01, or *p* < 0.05), whereas in BT474 cells, only H-SLC shows an inhibitory effect (*p* < 0.05). The migratory capacity of SLC was evaluated using a transwell assay. BT474 breast cancer cells grow very slowly, and many cells fail to migrate, so subsequent experiments only examined the migration of HCC1954 cells. HCC1954 breast cancer cells exhibited a significant increase in migratory activity under both normoxic and hypoxic conditions. The data in Figure 2G indicated that treatment of HCC1954 cells with varying concentrations of SLC led to a significant decrease in their migratory ability compared to the Ctrl group in HCC1954 cells. Collectively, these findings suggest that SLC effectively inhibits both the proliferation and migration of breast cancer cells, with a more pronounced effect observed under normoxic conditions.

### 2.3. Glucose Uptake, Intracellular ATP Content, Real-Time ATP Production Rate and Oxygen Consumption Rate (OCR)

**Figure 3:**
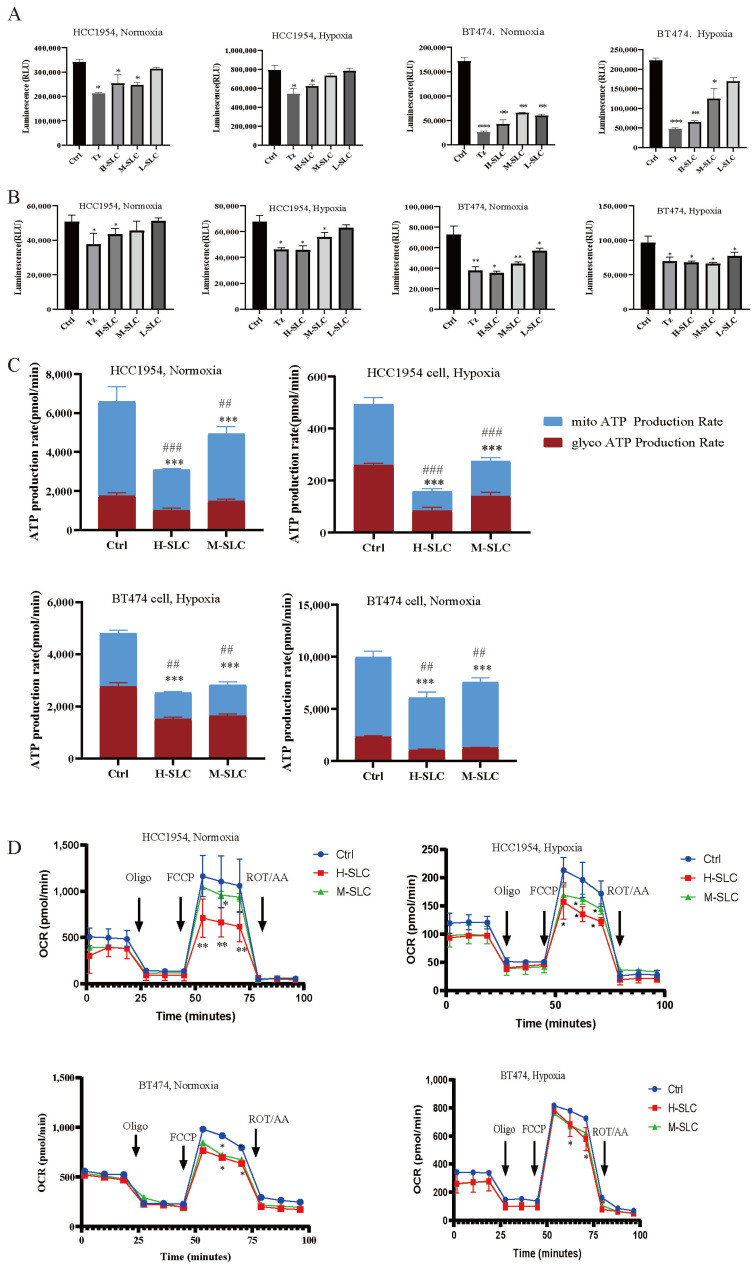
Effect of SLC on glucose uptake, intracellular ATP content, real-time ATP production rate and Oxygen Consumption Rate on HCC1954 and BT474 breast cancer cells under normoxic and hypoxic conditions. (**A**) Glucose uptake. (**B**) Intracellular ATP content. (**C**) Mitochondrial ATP and GlycoATP production rates. (**D**) OCR value indicated Oxygen Consumption Rate. N = 3. Mitochondrial ATP production rates were compared with Ctrl group, * *p* < 0.05, ** *p* < 0.01, *** *p* < 0.001. GlycoATP production rates were compared with Ctrl group, ^##^ *p* < 0.01, ^###^ *p* < 0.001.

Glucose uptake was significantly impacted in BT474 and HCC1954 cells following SLC therapy, both under normoxic and hypoxic conditions. As shown in Figure 3A, it was reduced after treatment with SLC in normoxic environment in BT474 and HCC1954 cells after 24 h, especially the H-SLC, but slightly diminished in hypoxic environment. Intracellular ATP levels were observed to decrease significantly following SLC treatment under both normoxic and hypoxic conditions, except at L-SLC and M-SLC, which did not elicit a significant effect in HCC1954 cells, as illustrated in Figure 3B. Subsequently, mitochondrial and glycolytic ATP production rates were evaluated using the Seahorse XF ATP rate assay, with the findings depicted in Figure 3C. After being treated with SLC for 24 h, both mitoATP and glycoATP production rates were significantly inhibited (*p* < 0.001) in two cell lines under normoxic conditions, which was compared with the Ctrl group. Meanwhile, under hypoxic conditions, after being treated with SLC for 24 h, the H-SLC and M-SLC groups both significantly inhibited the mitoATP and glycoATP production rates in BT474 and HCC1954 cells which compared with the Ctrl group (*p* < 0.001). We subsequently tested OCR data, which represented the measured values of mitochondrial oxidative phosphorylation. As shown in Figure 3D, the OCR values were both decreased by H-SLC and M-SLC in BT474 and HCC1954 cells under normoxic conditions. In a hypoxic environment, compared to the Ctrl group, the OCR values were significantly inhibited only by H-SLC in two breast cancer cells. The results indicated that SLC reduced the oxygen consumption rate, indicating impaired mitochondrial oxidative phosphorylation in BT474 and HCC1954 cells.

### 2.4. Detection of Mitochondrial Reactive Oxygen Species (mtROS), Mitochondrial Membrane Potential (MMP) and Intracellular Reactive Oxygen Species (ROS)

**Figure 4:**
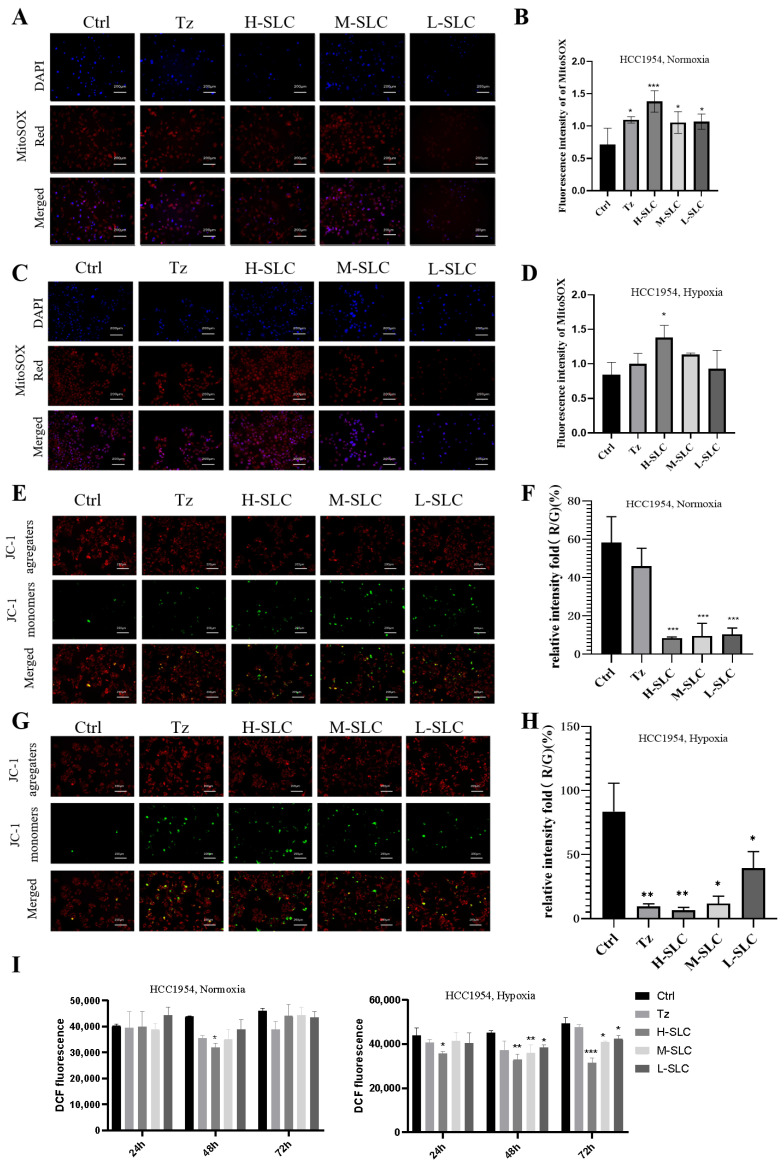
Effect of SLC on mtROS, MMP and intracellular ROS on HCC1954 breast cancer cells under normoxic and hypoxic conditions. (**A**,**C**): The fluorescence staining of mtROS by Mito SOX Red kit. DAPI (4′,6-diamidino-2-phenylindole)-stained nuclei appear in blue, Mito SOX-stained mtROS appear in red. 20 μm. (**B**,**D**): Analyze fluorescence intensity using Image J 1.8.0 software. (**E**,**G**): The MMP was measured by JC-1 staining. J-aggregates exhibit red staining, whereas J-monomers display green staining. (**F**,**H**): The Red/Green relative intensity of mtROS was calculated by Image J software. (**I**) Intracellular ROS levels were measured. N = 3. Compared with Ctrl group, * *p* < 0.05, ** *p* < 0.01, *** *p* < 0.001.

Given that mitochondria are the primary source of reactive oxygen species (ROS) within cells, the increased production of ROS is associated with oxidative stress, which can result in cellular damage [32]. The Mito SOX Red kit is used to measure mitochondrial ROS release. As shown in Figure 4A–D, there was a significant increase in SLC groups under normoxic and hypoxic conditions, compared with the Ctrl group (*p* < 0.05 and *p* < 0.001), which will cause progressive damage to mitochondrial membrane phospholipids, DNA and proteins. The mitochondrial membrane potential (MMP) was assessed using JC-1 staining, with results quantified by the ratio of red to green fluorescence; a lower ratio indicates more severe MMP damage [33]. Representative fluorescence images of the MMP are presented in Figure 4E,G, analysis of fluorescence values revealed that the red/green fluorescence ratio in the Tz and SLC treatment groups was lower than that observed in the control groups, as illustrated in Figure 4F,H. This outcome implies that SLC possesses a significant capacity for energy generation. Intracellular ROS levels were quantified using the DCFH-DA probe in Figure 4I, and the data indicated that neither Tz nor SLC significantly affected intracellular ROS levels under normoxic conditions; however, a slight reduction was observed under hypoxic conditions at 48 and 72 h.

### 2.5. SLC Inhibited the HER2 Signaling Pathway

**Figure 5:**
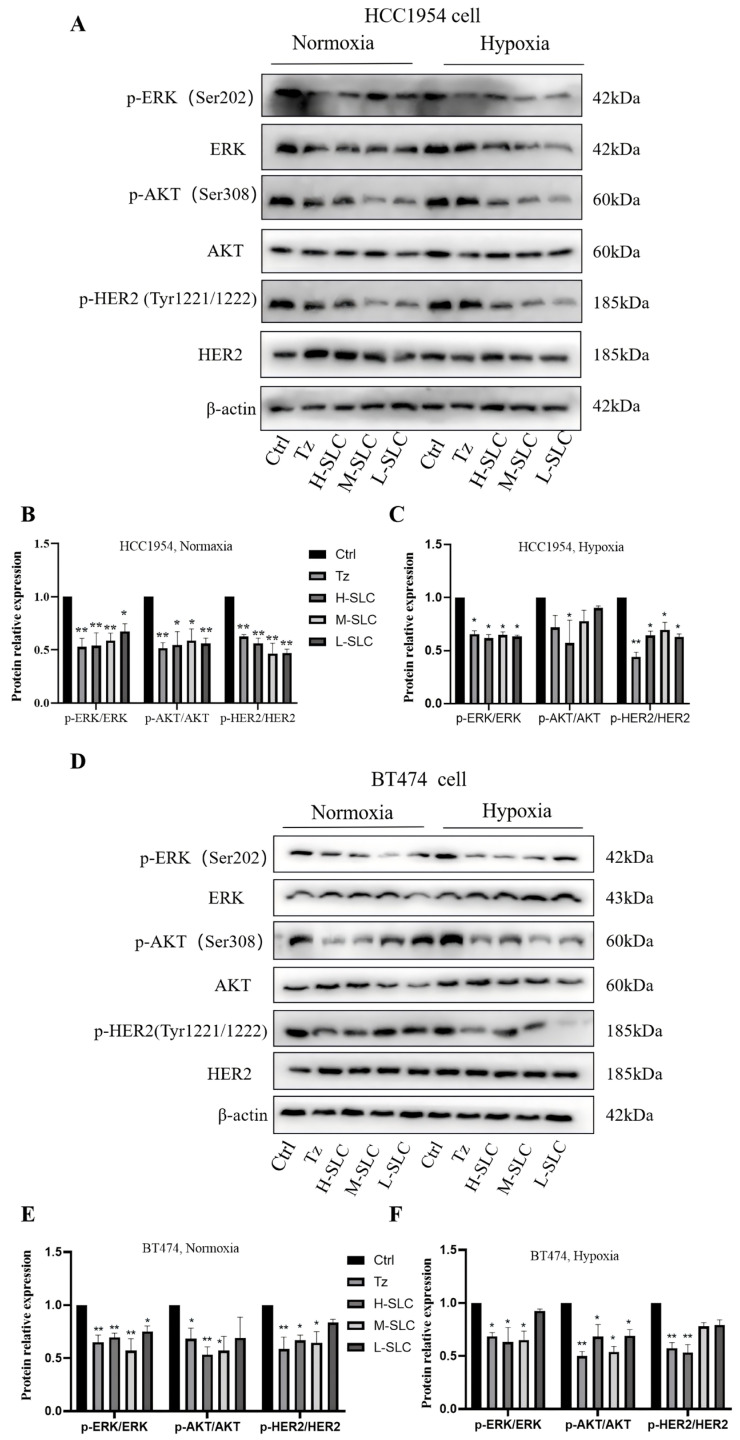
SLC inhibited the HER2 signal pathway via inhibiting the levels of p-HER2/HER2, p-AKT/AKT and p-ERK/ERK on HCC1954 and BT474 cells. (**A**) The protein expression of p-HER2, HER2, p-AKT, AKT, p-ERK and ERK under normoxic and hypoxic conditions on HCC1954 cells; (**D**) The protein expression of p-HER2, HER2, p-AKT, AKT, p-ERK and ERK under normoxic and hypoxic conditions on BT474 cells; (**B**,**C**,**E**,**F**): The protein relative expression was analyzed by Image lab 6.0 software. N = 3. Compared with Ctrl group, * *p* < 0.05, ** *p* < 0.01.

The accuracy assessment of HER2 status is crucial for predicting the response to HER2-targeted therapies in breast cancer [34]. To investigate whether SLC has an inhibitory effect on the HER2 signaling pathway, we will examine the impact of SLC on the expression of key proteins within this pathway [3]. The results indicated that Tz, H-SLC, and M-SLC significantly inhibited the levels of p-HER2/HER2, p-AKT/AKT, and p-ERK/ERK in HCC1954 and BT474 cells under normoxic conditions. Consistent with these findings, under hypoxic conditions, Tz, H-SLC and M-SLC also suppressed the levels of p-HER2/HER2, p-AKT/AKT, and p-ERK/ERK; however, L-SLC exhibited no significant efficacy (Figure 5). These results suggest that SLC suppresses the HER2 signaling pathway in breast cancer cells under both normoxic and hypoxic conditions.

### 2.6. SLC Inhibited the Activation of PDK1 to Suppress Mitochondrial Oxidative Phosphorylation in HCC1954 Breast Cancer Cells

**Figure 6:**
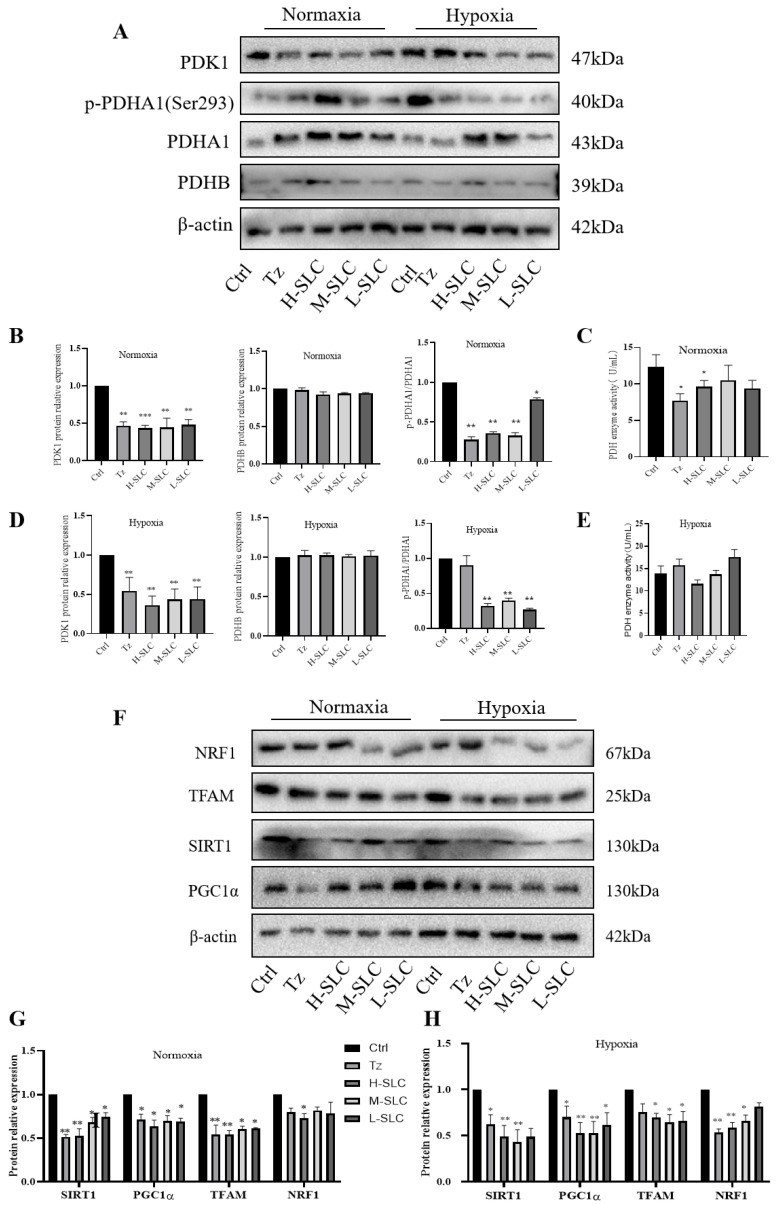
Effects of SLC on the expression of proteins within the PDK1/PDHA1 and SIRT1/PGC-1α/NRF1/TFAM signaling pathways on HCC1954 breast cancer cells. (**A**): PDK1, p-PDHA1, PDHA1and PDHB protein band under normoxic and hypoxic conditions; (**C**,**E**): PDH enzyme activity under normoxic and hypoxic conditions; (**B**,**D**,**G**,**H**): The protein relative expression was analyzed by Image J software; (**F**): NRF1, TFAM, SIRT1 and PGC1α protein band under normoxic and hypoxic conditions. N = 3 Compared with Ctrl group, * *p* < 0.05, ** *p* < 0.01, *** *p* < 0.001.

PDK1 is recognized as a critical regulator of glycolysis and serves as a targetable determinant of various metabolic states in cancer [35]. In normoxic conditions and hypoxic conditions, as illustrated in Figure 6A–C, SLC was found to reduce the protein expression of PDK1 and inhibit the phosphorylation of PDHA1 at Ser293, consequently repressing pyruvate dehydrogenase (PDH) activity, while the expression of PDHB remained unchanged. The inhibition of PDK1 leads to a reduction in the phosphorylation of PDHA1 at Ser293, lowers the rate of mitochondrial oxidative phosphorylation, and inhibits overall oxidative phosphorylation. Notably, PDH enzyme activity was significantly downregulated by H-SLC under normoxic conditions, with a lesser degree of reduction observed under hypoxic conditions, as shown in Figure 6C,E.

It is well established that PDK1 impairs mitochondrial function and facilitates the transition of glucose metabolism from mitochondrial oxidative phosphorylation to aerobic glycolysis [36]. The transcriptional coactivator PGC-1α plays a pivotal role in maintaining energy metabolism homeostasis and is closely associated with mitochondrial function. The SIRT1-dependent PGC-1α/NRF1/TFAM signaling pathway is integral to mitochondrial biogenesis and function [37,38]. We assessed the impact of PDK1 on protein expression within the SIRT1/PGC-1α/NRF1/TFAM signaling axis. As demonstrated in Figure 6F,G, under normoxic conditions, the protein expressions of SIRT1, PGC-1α, and TFAM in the SLC group were significantly downregulated compared to the Ctrl group, while NRF1 protein expression was inhibited solely in the H-SLC groups. Similarly, the expression of these four proteins was also suppressed under hypoxic conditions, as shown in Figure 6F,H. These findings indicate that SLC reduces the expression levels of PDK1 and exerts a substantial inhibitory effect on mitochondrial function through the SIRT1/PGC-1α/NRF1/TFAM signaling axes, thereby regulating mitochondrial oxidative phosphorylation in HCC1954 cells.

### 2.7. SLC Inhibited PDK1, Inducing Apoptosis in HER2-Positive HCC1954 Breast Cancer Cells

**Figure 7:**
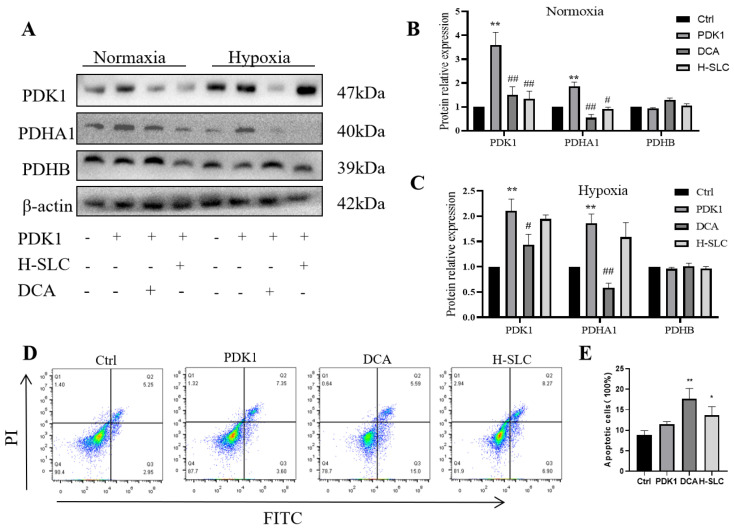
SLC inhibited PDK1 to induce cell apoptosis in HER2-positive HCC1954 breast cancer cells. (**A**). The protein expression of PDK1, PDHA1 and PDHB after overexpression of PDK1; (**B**,**C**). The analysis of WB band by Image Lab software. (**D**). SLC induces cell apoptosis after overexpression of PDK1 by flow cytometry; (**E**). The flow data analysis by flowjo10.0 software. N = 3. Compared with Ctrl group, * *p* < 0.05, ** *p* < 0.01. Compared with PDK1 overexpress group, ^#^ *p* < 0.05, ^##^ *p* < 0.01.

To further investigate the proposed role of PDK1 in inhibiting pyruvate dehydrogenase (PDH) activity, we measured the phosphorylation of PDH, which was inhibited by PDK1. In Figure 7A–C, following PDK1 overexpression, the protein expression of PDHA1, but not PDHB, was upregulated in HCC1954 cells. Treatment with H-SLC and DCA resulted in a reduction in PDK1 and PDHA1 protein expression (*p* < 0.01 and *p* < 0.05), while PDHB expression remained unaffected. Additionally, we examined the impact of SLC treatment on PDK1, PDHA1, and PDHB under hypoxic conditions, but no significant changes were observed. However, PDK1 expression was found to be upregulated under hypoxic conditions compared to normoxic conditions. This finding indicates that once a hypoxic microenvironment has been established within the tumor, the therapeutic efficacy of SLC may be limited. We then aimed to determine whether PDK1 is involved in cell proliferation rates and apoptosis [39]. To explore this, we overexpressed PDK1 and assessed the apoptosis rate of SLC using flow cytometry. As shown in Figure 7D,E, SLC and DCA significantly increased the apoptosis rate (*p* < 0.01 and *p* < 0.05). These results indicate that SLC represses the protein expression of PDK1 and PDHA1 and induces apoptosis following PDK1 overexpression.

### 2.8. SLC Inhibits Tumor Growth and Induces Cell Apoptosis in the HCC1954 Xenograft Model

**Figure 8:**
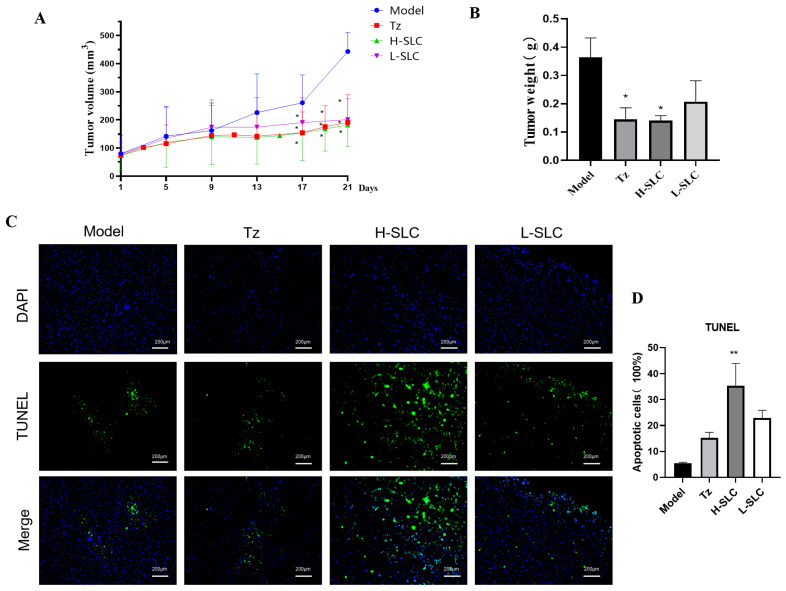
SLC inhibits tumor growth, induces cell apoptosis in HCC1954 xenograft model. (**A**) Tumor volume. (**B**) Tumor weight. (**C**) Cell apoptosis using TUNEL staining. Fluorescein isothiocyanate (FITC) labels apoptotic cells in green, whereas 4′,6-diamidino-2-phenylindole (DAPI) stains the nuclei in blue. (**D**) Calculate apoptosis rate using by Image J software. N = 6. Tz (15 mg/kg, administered weekly), H-SLC (6.32g·kg^−1^·d^−1^), L-SLC (3.16 g·kg^−1^·d^−1^). Compared with Model group, * *p* < 0.05, ** *p* < 0.01.

To further elucidate the anticancer properties of SLC, we established HCC1954 xenografts in nude mice and administered treatments of normal saline (Ctrl), SLC (30 mg/kg), or T-mab (30 mg/kg). After a treatment period of 21 days, SLC significantly decreased tumor volume (*p* < 0.05) compared to the control group (Figure 8A). Both Tz and H-SLC also resulted in reduced tumor weight, with the H-SLC group exhibiting a significantly lower tumor weight than the model group (*p* < 0.05) (Figure 8B). To investigate the molecular mechanisms underlying the tumor-inhibitory effects of SLC, we assessed its impact on tumor apoptosis. DAPI and TUNEL staining techniques were utilized to differentiate apoptotic cells from total cells. The H-SLC and L-SLC groups displayed a higher number of apoptotic cells compared to the model group following 21 days of SLC treatment (Figure 8C). The proportion of apoptotic cells in the H-SLC group reached 40%, which was significantly greater than that observed in the L-SLC group (*p* < 0.05) (Figure 8D).

### 2.9. SLC Repress HER2 Signaling Pathway, Reduce PDK1 and Regulate SIRT1/PGC-1α/NRF1/TFAM Signaling in HCC1954 Xenografts Model

**Figure 9:**
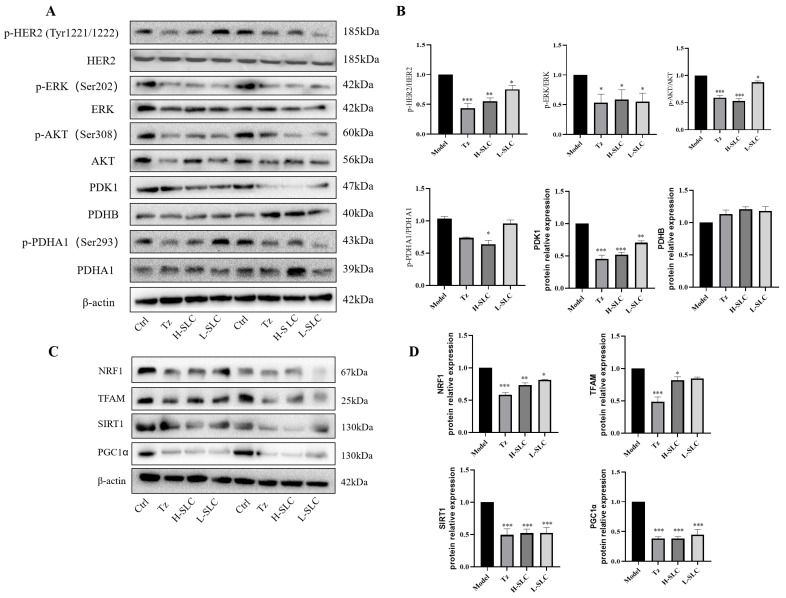
SLC repressed HER2 signal pathway, inhibited PDK1/PDHA1 and SIRT1/PGC-1α/NRF1/TFAM signaling pathways in HCC1954 xenograft model. (**A**) The expression of key proteins involved in three signaling pathways in tumors. (**B**,**D**): Calculate protein expression levels by Image Lab software. (**C**) The protein expression of NRF1, TFAM, SIRT1 and PGC1α in tumor. N = 6. Compared with Model group, * *p* < 0.05, ** *p* < 0.01, *** *p* < 0.001.

Anti-HER2-targeted therapy has emerged as a principal strategy in the treatment of HER2-positive breast cancer [40]. We showed that the level of p-HER2/HER2, p-ERK/ERK, p-AKT/AKT were all decreased in trastuzumab and SLC groups (Figure 9A,B). Next, we examined the protein expression of PDK1, PDHB, PDHA1 and p-PDHA1 in the xenograft model by Western blot. Consistent with our findings in vitro, trastuzumab and SLC treatments significantly reduced the level of PDK1, PDHB and p-PDHA1/PDHA1 (Figure 9C,D). In further, SLC downregulated the protein expression of SIRT1, PGC-1α, NRF1 and TFAM, which demonstrated the role of SLC in inhibiting SIRT1/PGC-1α/NRF1/TFAM signaling axis. These results indicated that SLC significantly not only inhibited the HER2 signaling pathway but also modulated PDK1 and SIRT1/PGC-1α/NRF1/TFAM signaling pathway in HCC1954 xenograft model in vivo.

### 2.10. Molecular Docking and Molecular Dynamics of Bioactive Compounds from SLC on the Essential Target Protein PDK1

Molecular docking is primarily utilized to assess the spatial complementarity and binding energy between ligands and receptors. In order to investigate the docking interactions of the active constituents of SLC with key molecular targets, we selected PDK1, which exhibited significant intersection target survivals, along with the 113 active compounds derived from SLC (refer to Supplementary File S1, Table S1). The corresponding crystal structure of PDK1 (PDB ID: 5HKM) was retrieved from the Protein Data Bank (PDB). The three-dimensional configurations of the active compounds were obtained from PubChem in SDF format, subsequently converted to mol2 format files, and then subjected to molecular docking using Sybyl 2.0. A total score of ≥5 indicates a favorable binding affinity between the active ingredient and the target protein, while a total score exceeding 7 signifies a very strong binding activity. Based on the data acquired, we established a threshold for docking results at a total score of ≥5 [41]. For comprehensive molecular docking data, please refer to Supplementary File S2, Table S2. The experimental findings revealed that 46 key active ingredients targeted the PDK1 protein, with 17 of these active ingredients demonstrating a total score of ≥7. The active ingredients identified include monomethyl lithospermate, icariside F2, ethyl lithospermate, l-alpha-Palmitin, satollinoleic acid 1, tetramethylpyrazine, daidzin, tilianin, acacetin-7-O-alpha-L-rhamnopyranoside, angelicide, neocarthamin, lithospermic acid, chuanxiongoside A, astragalin-1, zoomaric acid, and daturic acid. Notably, the highest docking score was observed for monomethyl lithospermate. Monomethyl lithospermate emerged as the most active compound, achieving a total score of 10.2779. The report indicated that monomethyl lithospermate is derived from the extract of *Salvia miltiorrhiza*, which has been shown to inhibit cervical cells [42]. Additionally, Monomethyl lithospermate also protects oxygen glucose deprivation/reoxygenation-induced SHSY-5Y cells in vitro via activation of PI3K/Akt signaling [43]. The second active ingredient was icariside F2, which is a potent NF-κB inhibitor and has anti-inflammatory activity [44].

**Figure 10:**
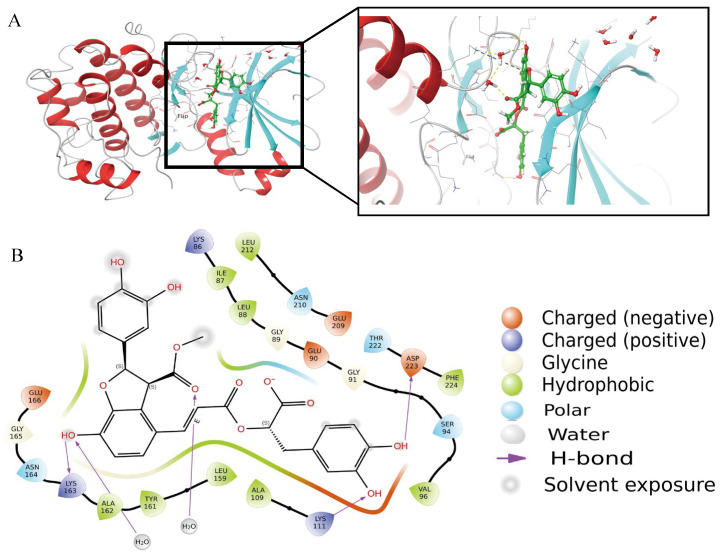
A schematic of molecular docking. (**A**). The overall binding figure with PDK1. (**B**). High activity binding site.

Figure 10A presents a schematic representation of molecular docking. The docking score recorded was −6.36664 kcal/mol, suggesting a robust affinity for binding to the target PDK1 [45]. Figure 10B showed the hydroxyl group on the left end of the ligand first forms a direct bond with LYS163 and then connects to the main chain through a single water molecule bridge at the central carbonyl, creating a stable dynamic lock. The hydroxyl group on the right end forms a classic donor-acceptor geometric relationship with ASP223, while the hydroxyl group adjacent to the aromatic ring also exhibits a highly directional hydrogen bond with LYS111. The hydrophobic layers are tightly constructed by LEU88, LEU159, LEU212, VAL96, and PHE224 around the framework, maximizing the burial of the ligand’s hydrophobic surface and reducing solvent accessibility. Three acidic residues, GLU90, GLU166, and GLU209, are distributed along the edge of the pocket; their negatively charged environment not only provides long-range electrostatic compensation with the ligand’s polar groups.

These result indicates that Monomethyl lithospermate exhibits a stronger binding affinity for the target protein PDK1. The SLC component may be regarded as a promising lead compound for the development of anti-breast cancer pharmaceuticals, demonstrating favorable prospects for further advancement.

## 3. Discussion

Cancer cells demonstrate increased glycolytic activity relative to normal cells, which has led to the prevailing assumption that oxidative phosphorylation (OXPHOS) is universally downregulated across all cancer types. Nonetheless, recent research has revealed that OXPHOS can be upregulated in specific cancers, including breast cancer, melanoma, and glioma, among others [46]. An analysis of gene expression profiles from 2000 breast cancer patients identified a significant transcriptional upregulation of OXPHOS [47]. Furthermore, transcriptomic analyses and Western blot assays have confirmed pronounced OXPHOS upregulation in breast cancers lacking RB1 expression [48]. Currently, the repertoire of OXPHOS inhibitors undergoing preclinical or clinical investigation remains limited. Therefore, the discovery and development of OXPHOS-targeted inhibitors constitute a largely unexplored yet promising strategy in cancer therapeutics [49]. These findings indicate that OXPHOS represents a potential therapeutic target in breast cancer.

Nowadays, numerous traditional Chinese medicine monomer inhibitors have been identified that inhibit OXPHOS, including Demethylzeylasteral [50], the extract of Cortex Periplocae, known as Periplocymarin (PPM) [51], and a novel peptide inhibitor of C1QBP that impairs mitochondrial function and targets triple-negative breast cancer [52]. In further, SLC decreased intracellular ATP, reduced intracellular ROS levels, inhibited both mitoATP and glycoATP production rates, and especially decreased the OCR values. These data showed SLC not only inhibits glycolysis but also OXPHOS in HER2-positive cancer cells.

Pyruvate dehydrogenase kinases (PDKs) are serine/threonine kinases that play a pivotal role in the metabolic reprogramming of cancer cells, thereby contributing to tumor aggressiveness and therapeutic resistance. The present study demonstrates that inhibition of the PDK/PDHA1 signaling axis results in metabolic and redox dysregulation within cancer cells, ultimately inducing apoptotic cell death [53]. To date, several potent small-molecule inhibitors targeting PDKs have marked important advancements in cancer research. Notably, dichloroacetic acid (DCA) and its derivatives have exhibited significant antiproliferative effects across various cancer cell lines and murine models. However, these compounds have not successfully advanced through clinical trials, primarily due to off-target activities, limited potency, and inadequate selectivity [54]. Accordingly, the past two decades have witnessed focused efforts to develop novel therapeutics that overcome the pharmacokinetic limitations, suboptimal potency, and selectivity issues associated with DCA [55]. In our experimental findings, treatment with SLC resulted in a marked reduction in the expression levels of PDK1 and phosphorylated PDHA1 proteins relative to the Ctrl group. These results are consistent with previous findings [56,57]. Furthermore, SLC significantly inhibited PDH activity. Conventional understanding posits that a reduction in PDK1 and phosphorylated PDHA1 (p-PDHA1) levels would result in an enhancement of PDH activity; however, our experimental findings indicate a decrease in PDH activity. This discrepancy can be elucidated as follows: The mitochondrial pyruvate dehydrogenase complex (PDC) plays a pivotal role in the metabolic transition from the tricarboxylic acid (TCA) cycle and oxidative phosphorylation (OXPHOS) to aerobic glycolysis. This metabolic shift is predominantly regulated by four isoenzymes of pyruvate dehydrogenase kinase (PDK1-4) [58]. Notably, PDK3 expression is elevated in breast cancer tissues, akin to PDK1, and is induced by HIF-1α, exhibiting increased expression levels. PDK3 was found to have an enhanced level in BC tissues, which was similar to PDK1, which was induced by HIF-1a, and higher expression [22,59]. Consequently, it is insufficient to evaluate PDK1 in isolation; assessment of PDK3 is also imperative. An upregulation of PDK3 would contribute to the suppression of PDH activity. Furthermore, under pronounced hypoxic conditions, forced PDK1 expression in hypoxic HIF-1alpha null cells increases ATP levels, attenuates hypoxic ROS generation, and rescues these cells from hypoxia-induced apoptosis [19].

The suppression of mitochondrial biogenesis constitutes a strategic approach to reducing OXPHOS. PGC-1α is a pivotal regulator essential for mitochondrial biogenesis. PGC-1α activates nuclear respiratory factor 1 (NRF1) and nuclear respiratory factor 2 (NRF2), which in turn facilitate the synthesis of mitochondrial transcription factor A (TFAM) [60,61]. Accordingly, the inhibition of mitochondrial biogenesis has been proposed as a novel therapeutic strategy in oncology [62]. Various small-molecule inhibitors have been developed to target key regulators involved in tumor mitochondrial biogenesis, including PGC-1α, PGC-1β, the estrogen-related receptor family (ERRs), and estrogen receptor alpha (ERα) [63]. For example, BRAF inhibitors have been shown to induce PGC-1α, thereby promoting mitochondrial biogenesis and leading to increased reliance on OXPHOS. Our findings demonstrate that SLC reduces the protein expression levels of SIRT1, PGC-1α, NRF1, and TFAM. These data suggest that SLC effectively suppresses the SIRT1/PGC-1α/NRF1/TFAM signaling pathway, resulting in the downregulation of OXPHOS in HER2-positive cancer. Collectively, these findings suggest that SLC disrupts cellular energy metabolism and impairs mitochondrial functional status. Nevertheless, it remains unclear whether these mitochondrial effects result from a direct action of the drug on mitochondria or represent an indirect adaptive response secondary to glucose deprivation.

In the further, we focus on the molecular docking and dynamics of bioactive compounds from SLC with the target protein PDK1. PDK1 was chosen due to its significant intersection target survivals, and its crystal structure (PDB ID: 5HKM) was obtained from the Protein Data Bank. A total of 113 active compounds from SLC were analyzed, and 46 active ingredients were found to target PDK1, with 17 achieving a score of ≥7. Key active ingredients identified include: Monomethyl lithospermate (highest score of 10.2779), Icariside F2 (potent NF-κB inhibitor). The docking score for PDK1 was −6.36664 kcal/mol, indicating strong binding affinity. The docking analysis revealed specific interactions between the ligand and PDK1, including hydrogen bonds and hydrophobic interactions with various amino acids.

There are still several issues in the article that need to be addressed further. First, the paper indicates that HER2-positive breast cancer primarily relies on glycolysis [64,65]. However, it has been reported that breast cancer stem cells (BCSCs) depend more on oxidative phosphorylation for their energy needs [66]. Traditional HER2-targeted therapies may effectively eliminate the majority of differentiated cancer cells; however, they often fail to eradicate tumor stem cells, which can result in disease relapse. The combined inhibition of HER2 and oxidative phosphorylation (OXPHOS) presents a promising strategy for targeting tumor stem cells, potentially leading to more sustained therapeutic outcomes. Second, it is evident that hypoxia induces a shift in ATP production from glycolysis to mitochondrial respiration [67], and hypoxia promotes the expression of PDK1 [18]. Our data show that PDK1 is expressed under both normoxic and hypoxic conditions, with higher levels observed in hypoxic tumor tissue. Currently, there are no reports addressing the effects of hypoxia on the phosphorylation of PDK1 and PDHA, nor elucidating the underlying biological mechanisms. These aspects warrant further comprehensive investigation. Third, understanding the regulation of HIF activity across various breast cancer subtypes is crucial, as hypoxic conditions up-regulated GLUT1 mRNA expression, increased the HER2 membrane protein fraction, and also there is a significant interplay between HER2 expression and HIF-2α in breast cancer [68]. and highlights the potential of targeting HIF-2α as a therapeutic strategy for HER2-positive breast cancer [69]. Therefore, targeting the inhibition of HIF activity and HER2 expression may be more beneficial for breast cancer treatment, but the underlying mechanisms still need to be further elucidated. Fourth, future investigations employing permeabilized cell systems or models utilizing alternative fuel sources are warranted to elucidate the drug’s precise molecular targets within mitochondria. In the end, SLC, a traditional Chinese medicine formulation, comprises numerous identified active constituents. Molecular docking screening was employed to identify certain components; subsequently, the stability of the interactions between PDK1 and these components was evaluated through analyses of root-mean-square deviation (RMSD) and root-mean-square fluctuation (RMSF). These findings were further corroborated by in vitro and in vivo experimental validations.

## 4. Materials and Methods

### 4.1. Chemicals and Reagents

*Salvia miltiorrhiza* Bunge (Dan Shen, lot number: 230312), *Ligusticum wallichii* Franch (Chuang Xiong, lot number: 220916), *Carthamus tinctorius* L. (Hong Hua, lot number: 230129) were purchased from Jiangxi Traditional Chinese Medicine Decoction Co., Ltd. (Jiujiang, China), and were authenticated by Professor KZ Deng in Jiangxi University of Chinese Medicine. Trastuzumab (Tz, J20181017) was acquired from Shanghai Roche Pharmaceuticals (Shanghai, China). Bovin serum albumin (BSA) (KC020-01) was purchased from Shanghai Juding Biotechnology Co., Ltd. (Shanghai, China). CellTiter-Lumi™ Steady Plus Luminescent Cell Viability Assay Kit, Reactive Oxygen Species Detection Kit, Mitochondrial Membrane Potential Assay Kit (JC-1), RIPA lysis buffer, TUNEL Apoptosis Detection Kit were purchased from Beyotime (Shanghai, China). MitoSOX Red was purchased by MCE (Monmouth Junction, NJ, USA). PVDF membranes was purchased by Merck Millipore (Billerica, MA, USA). PDK1 (Abcam, Cambridge, UK, Ab207450), p-PDHA1 (Abcam, ab177461), PDHA1 (Abcam, ab168379), AKT (Proteintech, Rosemont, IL, USA, 10176-2-AP), p-AKT (S308) (CST, 13038T), ERK (Proteintech, 11257-1-AP), p-ERK (Thr202) (CST, 4370T), AKT (Proteintech, 10176-2-AP), p-HER2/ErbB2 (Tyr1221/1222) (CST, 2243T), HER2/ErbB2(CST,4290T), SIRT1 (Proteintech, 60303-1-Ig), PDHB (Proteintech, 14744-1-Ap), PGC-1α (CST, 2178s), NRF-1 (Proteintech, 12482-1-Ap), TFAM (Proteintech, 22568-1-AP). Overexpression PDK1 plasmid was purchased from Genechem (Shanghai, China), Opti MEM medium was purchased from GIBCO Corporation (Waltham, MA, USA), Lipofectamine^®^ 3000 Transfection Kit from Invitrogen was acquired from Thermo Fisher Scientific (Shanghai, China). ECL reagent (CW0049M) was purchased by CWBIO (Nanjing, China). Overexpression of PDK1 and the control vector were procured from Gene Biotechnology (Shanghai, China). Glucose Uptake-Glo™ Assay (#J1342) was obtained from Promege (Madison, WI, USA). Seahorse XF Real-Time ATP Rate Assay kit (#103591-100) was obtained from Agilent (Santa Clara, CA, USA). 2-Dexyl-D-glucose (2DG) was purchased from Sigma (Shanghai, China). HER2-positive breast cancer cells (TD474 and HCC1954) were obtained from the Cell Bank, Type Culture Collection of the Chinese Academy of Sciences (Shanghai, China).

### 4.2. Preparation of Herbal Formulations Extracts

Prior to the extraction process, the herbs were ground into a fine powder. The formulation of the SLC recipe consisted of an equal ratio of *Salvia miltiorrhiza* Bunge (Dan Shen), *Ligusticum wallichii* Franch. (Chuang Xiong), and *Carthamus tinctorius* L. (Hong Hua) in a 1:1:1 ratio. A total of 1 kg of these powdered samples was subjected to reflux extraction using 10 L of water (1:10, *w*/*v*) for a duration of 2 h, followed by a second extraction for an additional 2 h. The combined supernatants from the two extraction cycles were subsequently concentrated under reduced pressure at a temperature of 60 °C and subsequently lyophilized. The resulting product was sealed and stored at a temperature of −20 °C.

### 4.3. Characterization of SLC via UHPLC-Q-TOF/MS

The Waters SYNAPT G2-Si mass spectrometer (Waters Corporation, Milford, MA, USA) was utilized to obtain raw data via ultra-high-performance liquid chromatography coupled with quadrupole time-of-flight mass spectrometry (UHPLC-Q-TOF-MS). The analysis employed a Waters ACQUITY UPLC HSS T3 analytical column (2.1 mm × 100 mm, 1.8 μm particle size) alongside a Waters ACQUITY UPLC HSS T3 pre-column (2.1 mm × 5 mm, 1.8 μm particle size). The mobile phase comprised water containing 0.1% (*v*/*v*) formic acid (solvent A) and acetonitrile (solvent B). The gradient elution was programmed as follows: 2% B from 0 to 1 min; 2% to 10% B from 1 to 7 min; 10% to 34% B from 7 to 20 min; 34% to 50% B from 20 to 30 min; 50% to 100% B from 30 to 37 min; 100% to 2% B from 37 to 38 min; and 2% B from 38 to 42 min. The column temperature was maintained at 40 °C, with a flow rate set at 0.3 mL/min. Electrospray ionization (ESI) parameters were configured as follows: ion spray voltage floating (ISVF) at +5000 V and −4500 V; ion source temperature at 400 °C; nebulizer gas pressure (GS1) at 50 psi; curtain gas (CUR) at 35 psi; auxiliary gas (GS2) at 50 psi; collision energy (CE) at ±30 V with a collision energy spread (CES) of ±15 V. Mass spectrometric data were acquired over a mass-to-charge ratio (*m*/*z*) range of 100 to 1000 Da in both positive and negative ionization modes. Data acquisition and processing were performed using PeakView software 1.2.

### 4.4. Cell Culture and Colony Formation

HER2-positive breast cancer cell lines BT474 (Luminal B/HR+/HER2+) and HCC1954 (HER2-Enriched/HR-/HER2+) were utilized in this study. The cell lines were cultured in RPMI-1640 medium supplemented with 10% heat-inactivated fetal bovine serum (FBS) and 1% Penicillin/Streptomycin, and maintained at 37 °C in a humidified atmosphere with 5% CO_2_. For the colony formation assay, BT474 cells (2 × 10^3^ cells) and HCC1954 cells (1.5 × 10^3^ cells) were seeded in 6-well plates. After a 10-day treatment with L-SLC, M-SLC, and H-SLC at concentrations of 625, 1250, and 2500 μg/mL, respectively, as well as trastuzumab (Tz) at 25 μg/mL, all maintained at 37 °C, the colonies were fixed using 4% paraformaldehyde. Subsequently, they were stained with a 1% crystal violet solution for 20 min. The colonies were then photographed and subjected to quantitative analysis.

### 4.5. Wound Healing and Cell Migration Assay

A wound healing assay was performed to assess the migratory capabilities of specific cell lines, namely BT474 and HCC1954. These cell lines were cultured at a density of 1 × 10^6^ cells per well in a 6-well plate. A 200 μL pipette tip was used to create linear scratches in the cell monolayer until approximately 90% confluence was achieved. The area of the wound was measured and recorded at both 0 and 24 h following the scratching procedure. Additionally, cell migration was assessed using a Transwell assay. A suspension of BT474 and HCC1954 cells (5 × 10^3^ cells per well) was placed in the upper chambers of the Transwell apparatus, while the lower chambers were filled with culture medium supplemented with 10% fetal bovine serum (FBS). The cells were incubated for 24 h under both normoxic and 1% hypoxic conditions. Subsequently, they were treated with varying concentrations of L-SLC, M-SLC, and H-SLC at concentrations of 625, 1250, 2500 μg/mL, respectively, and a positive control Tz (25 μg/mL), for an additional 24 h. After this incubation, cells that remained on the upper side of the membrane were carefully removed using a cotton swab. The cells were then fixed in 4% paraformaldehyde overnight, stained with 1% crystal violet for 20 min, and the number of migratory cells was quantified using an inverted light microscope.

### 4.6. Determination of Glucose Uptake, Intracellular ATP Content Assays and Pyruvate Dehydrogenase (PDH) Activity

Cancer cells were plated in six-well plates, and the culture medium was collected after a 24-h incubation period, during which the cells were treated with or without SLC (625, 1250, and 2500 μg/mL) for an additional 24 h. The concentration of glucose in the spent medium was quantitatively measured using the Glucose Uptake-Glo™ Assay kit. Additionally, the intracellular ATP content was assessed using the CellTiter-Lumi™ Steady Plus Luminescent Cell Viability Assay Kit. The pyruvate dehydrogenase (PDH) activity in both Ctrl and treated cells was evaluated using a Pyruvate Dehydrogenase assay kit. The membrane fraction was isolated from cell lysates and subsequently resuspended for the assay. PDH activity was quantified by measuring the optical density at 492 nm using a microplate reader. Prior to the analysis, a protein assay was conducted to determine the protein concentration of each sample.

### 4.7. Real-Time ATP Production Rate and Oxygen Consumption Rate (OCR)

The oxygen consumption rate (OCR), extracellular acidification rate (ECAR), and real-time ATP production rate were measured utilizing a Seahorse XFe96 Analyzer (Aligent, Santa Clara, CA, USA) in combination with the Seahorse XF Cell Mito Stress Test Kit, Seahorse XF Glycolytic Rate Assay Kit, and Seahorse XF Real-Time ATP Rate Assay Kit, respectively, in accordance with the manufacturer’s protocols. Specifically, breast cancer BT474 cells (5 × 10^4^ cells) and HCC1954 cells (7 × 10^4^ cells) were cultured overnight in custom XF24 microplates using 1640 medium in both normoxic and hypoxic incubators. After treated with or without L-SLC, M-SLC, H-SLC (625, 1250, and 2500 μg/mL) for an 24 h, the cells were washed with unbuffered 1640 medium and incubated in a CO_2_-free environment for one hour to acclimate to the assay medium. The following day, the cells were replaced with unbuffered RPMI-1640 assay medium and allowed to equilibrate for one hour. For the determination of OCR, sequential injections of 2 μM oligomycin, 0.4–0.5 μM FCCP, and 5 μM rotenone and antimycin A (Rote&AA) were administered. The absolute values of OCR were expressed in picomoles per minute. Data analysis was conducted using the Seahorse Wave Desktop Software 2.6.

### 4.8. Mitochondrial Reactive Oxygen Species (mt-ROS) Measurement

The assessment of cellular mitochondrial superoxide was performed using MitoSOX Red kit. Cells were cultured in a 6-multiwell plate at a density of 6 × 10^5^ cells per well and subjected to SLC treatment at three different concentrations (625, 1250, and 2500 μg/mL) for a duration of 24 h. Following treatment, the cells were washed with phosphate-buffered saline (PBS) and incubated with 2 µM MitoSOX Red dye for 60 min at 37 °C. After the incubation period, excess dye was removed by washing with PBS, and the cells were subsequently visualized using a fluorescence microscope, followed by analysis and quantification of fluorescence intensity utilizing Image J software [30].

### 4.9. Mitochondrial Membrane Potential (MMP) Assessment

The relative mitochondrial membrane potential was evaluated utilizing the JC-1 Mitochondrial Membrane Potential Assay Kit. Following treatment with L-SLC, M-SLC, H-SLC (625, 1250, and 2500 μg/mL) and Tz (25 μg/mL) or without, cells were collected and incubated with the JC-1 staining solution at 37 °C for a duration of 20 min. Fluorescence measurements were conducted using a flow cytometer (Beckman Coulter, Brea, CA, USA).

### 4.10. Intracellular Reactive Oxygen Species (ROS) Measurement

Cells (2 × 10^5^ cells/mL) were cultured in a 6-multiwell plate for 24 h and subjected to L-SLC, M-SLC, H-SLC (625, 1250, and 2500 μg/mL) and Tz (25 μg/mL) treatment for 24, 48, and 72 h, after which intracellular hydrogen peroxide (H_2_O_2_) levels, indicative of ROS, were evaluated by incubating the cells with H_2_DCFDA (20 μM) for 30 min at 37 °C. Fluorescence intensity was quantified using a fluorometer, with excitation set at 480 nm and emission at 530 nm.

### 4.11. Western Blot Analysis

Following treatment with L-SLC, M-SLC, H-SLC (625, 1250, and 2500 μg/mL) and Tz, proteins were extracted from cells or tissues utilizing RIPA lysis buffer. The proteins within the samples were separated using SDS polyacrylamide gel electrophoresis. Subsequently, the proteins were transferred onto PVDF membranes, which were then blocked with skim milk for one hour at ambient temperature. After the blocking step, the membranes were incubated overnight at 4 °C with primary antibodies diluted at a ratio of 1:1000. Then the PVDF membranes were washed three times with TBST. The corresponding secondary antibodies, diluted to 1:5000, were added and incubated for an additional hour. Detection of the antibody–antigen complexes was performed using an ECL reagent, and the resulting data were analyzed using Image J software.

### 4.12. PDK1 Overexpression Assay

Cells were trypsinized, quantified, and seeded into 6-well plates one day prior to transfection to achieve approximately 70% confluency at the time of transfection. PDK1 overexpression vector (pcDNA3.1-PDK1; Forward primer: CGCGGATCCATGAGGCTGGCGCGGCTGCTT; Reverse primer: CCGGAATTCCTAGGCACTGCGGAACGTCGT) and its corresponding control vector (pcDNA3.1) were prepared using a mixture containing 1 μg of plasmid DNA, 1 μL of P3000 reagent, and 50 μL of Opti-MEM serum-free medium, following the manufacturer’s protocol. After 72 h, the culture medium was replaced with fresh RPMI 1640 medium supplemented with 10% fetal bovine serum (FBS), 2500 μg/mL SLC, and 20 μM dichloroacetate (DCA) for an additional 48 h. Subsequently, the cells were harvested for verification by Western blot analysis. After 72 h, the viral culture medium was replaced with fresh 1640 containing 10% FBS and 2500 μg/mL SLC and 20 μΜ DCA for an additional 48 h, after which the cells were harvested for verification via Western blot analysis [11].

### 4.13. Xenograft Model in Nude Mice

A total of thirty-two female Balb/c nude mice, aged 4 to 6 weeks, were maintained in a specific pathogen-free environment. All experimental procedures received approval from the Institutional Animal Care and Use Committee and adhered to established institutional guidelines. The mice were subjected to the development of xenograft tumors through the subcutaneous injection of HCC1954 breast cancer cells (1 × 10^7^ cells) into their forelimbs. Upon reaching a tumor volume of 50 mm^3^, the mice were randomly assigned to one of four groups: Model, H-SLC (6.32 g·kg^−1^·d^−1^), L-SLC (3.16 g·kg^−1^·d^−1^), and a positive control group receiving Tz (15 mg/kg, administered weekly). SLC dosage was designed based on the reference of 18 g/day per medicinal ingredient specified in the Chinese Pharmacopoeia (2020 edition). Based on the dose conversion formula utilizing body surface area between humans and mice, the low and high doses for BALB/c mice were determined to be equivalent to one and two times the clinical dose, respectively. Mice in the control and model groups were treated with saline and SLC via gavage once daily, respectively. Body weight and tumor volume were monitored at three-day intervals. After a treatment period of twenty-one days, the mice were euthanized, and the tumors were excised and weighed. Tumor tissues were subsequently fixed in 4% paraformaldehyde or stored at −80 °C for further analysis via RT-PCR and Western blotting. The TUNEL assay was conducted using the TUNEL Apoptosis Detection Kit in accordance with the manufacturer’s instructions.

### 4.14. Molecular Docking and Analysis

The three-dimensional molecular structures of the compounds were optimized utilizing SYBYL 2.0 software (Shanghai Tri-I Biotech Inc., Shanghai, China). The homology model of the PDK1 protein was developed through the online software Swiss-Model (https://swissmodel.expasy.org/, accessed on 12 October 2025). The template protein crystal structure of PDK1 (PDB code: 5HKM) was downloaded from the RCSB Protein Data Bank (https://www.rcsb.org/, accessed on 9 August 2025). Molecular docking was performed using AutoDock Vina 1.1.2. The binding pattern models were generated using PyMOL 1.7.6, and the corresponding two-dimensional binding models were created using Discovery Studio 4.5. Critical interactions with the protein were illustrated using Pose Viewer and 2D interaction diagrams, while the binding conformations were visualized through Maestro to confirm stable and conserved binding patterns.

### 4.15. Statistical Analysis

Statistical analyses were performed using GraphPad Prism 8.0 (GraphPad Software, San Diego, CA, USA). Comparisons between two groups were conducted using Student’s *t*-test, while multivariate analyses were executed using one-way or two-way ANOVA. Results are presented as mean ± standard deviation (SD), with a significance threshold set at *p* value of less than 0.05.

## 5. Conclusions

SLC inhibited cell proliferation and migration, reduced glucose uptake and intracellular ATP levels, and decreased the production rates of mitoATP and glycoATP. Additionally, SLC diminished the mitochondrial membrane potential, intracellular ROS levels, and OCR values. Furthermore, SLC downregulated the levels of p-HER2/HER2, p-AKT/AKT, and p-ERK/ERK, thereby inhibiting the HER2 signaling pathway. Mechanistically, SLC decreased the expression levels of PDK1 and phosphorylated PDHA1, while also downregulating the protein expression of SIRT1, PGC-1α, NRF1, and TFAM. In summary, this study suggests that the herbal recipe SLC has the potential to act as an agent for inhibiting OXPHOS through the PDK1/PDHA1 and SIRT1/PGC-1α/NRF1/TFAM signaling axis to treat HER2-positive breast cancer.

## Data Availability

The original contributions presented in this study are included in the article/Supplementary Material. Further inquiries can be directed to the corresponding authors.

## Supplementary Materials

The following supporting information can be downloaded at: https://www.mdpi.com/article/10.3390/ijms262411970/s1.
