# Supplementary material for: Herbal Composition Inhibits Mitochondrial Oxidative Phosphorylation to Prevent HER2-Positive Breast Cancer and Identifies Potential Active Compounds
Source: Int J Mol Sci. 2025 Dec 12;26(24):11970. doi: 10.3390/ijms262411970 (PMC12733102; doi:10.3390/ijms262411970)
Supplement: Supplementary file 1 [file ijms-26-11970-s001.zip › Supplementary File S1.pdf]

Supplementary File S1

Table S1 Possible active ingredients form SLC by UHPLC-Q-TOF/MS

Number	Component name	Observed RT (min)	Formula	Adducts	Neutral mass (Da)	Observed m/z	Mass error (ppm)	MS/MS
1	(2E,8E)-tetradecadiene-4,6-diyne-1,11,14-triol	0.85	C14H18O3	+CH3COO	234.12559	293.1392	-0.7	171.05729、183.06269、185.05805、197.07670
2	neocarthamin	0.86	C12H18O2	+HCOO	450.11621	495.118	7.3	165.09146，193.08426，215.08088，245.16967，259.06568，267.02148，293.13876，495.12313
3	Methylcinnamate	0.88	C10H10O2	-H	162.06808	161.0597	-6.9	129.07050、159.08043
4	chuanxiongnolide L1	0.89	C10H16	-H	358.14164	357.1352	2.4	73.04091，101.09072，121.08096，157.08068，159.09624，161.07463，323.02779
5	6-o-syringyl-8-o-acetyl shanzhiside methyl ester	0.92	C28H36O16	+Cl	628.20034	663.1671	-3.9	429.15432、447.12565
6	Naringin	0.94	C27H32O14	-H	580.17921	579.1706	-2.3	57.08038，135.08093，161.09656，229.09154，297.12200，325.10545，475.14234，503.16999，579.17777
7	icariside F2	0.95	C18H26O10	+CH3COO	402.1526	461.163	-7.4	55.06261，59.05947，71.06143，73.04062，73.04062，87.05823，89.07435，99.09639，101.07527，107.08570，115.05483，117.07053，131.08660，147.08143，149.09658，161.09656，163.07541，189.05793，311.13586，327.12924，383.14807，399.14213，447.15277
8	l-Verbenone	0.96	C10H14O	-H	150.10447	149.097	-1.2	133.06598、149.09658、147.08143
9	hydroxysafflor yellow B	0.97	C29H38O15	+Na	626.22107	649.2089	-2.1	61.06308、73.06570、91.07816、103.07956、109.06829、145.08961、163.09989、451.18683
10	qt_carthamin	0.98	C43H42O22	+HCOO	620.11661	665.113	-2.8	59.05947，117.07053，223.09048，877.19864
11	Sitoindoside II	1.02	$C_{53}H_{92}O_7$	+HCOO	840.68431	885.6852	3.1	59.05947，71.06143，73.04062，225.10105，573.44510，885.68497

12	(+)ligusticumtone	1.25	C16H19O5	+K	291.12325	330.0851	-3.8	91.07857，163.10013
13	methyltanshinonate	1.44	C20H18O5	-H	338.11542	337.1071	-3.1	109.07972、251.12185
14	isoimperatorin	1.46	$C_{16}H_{14}O_4$	+Cl	270.08921	305.0557	-9.6	157.06299，173.05930，201.08982，225.09798，241.09343，305.05430
15	4-ethoxycinnamic acid	1.67	C11H12O3	-H	192.07864	191.0698	-8	121.04632、163.06547
16	dimethyllithospermate	2.8	C38H34O16	+HCOO	566.14243	611.1353	-8.8	133.06568、307.19000
17	3-(3-β-D-glucopyranosyloxy-butylidene)-7-hydroxy-phthalide	3.75	C21H20O12	+CH3COO	382.12638	441.1433	7.1	127.16528、201.16240、287.19768
18	2,4-Dichloro-5-([(E)-(4-chlorophenyl)methylidene]amino)benzoic acid	5.53	C5HCl3N2O	-H	168.0939	167.0856	-6	210.16806、223.16117
19	3R,8S,9Z-falcarindiol	6.31	C17H24O2	-H	260.17763	259.1721	6.9	239.19255、311.25844、367.21964
20	epidanshenspiroketallactone	7	C12H16O6	-H	284.14124	283.1358	6.4	139.05528，165.03250，167.04946，183.07904，209.05567，221.05540，223.12199，267.10795
21	benzyl-β-D-glucopyranoside	7.26	C20H24O6	+HCOO	270.11034	315.1078	-2.3	73.04326，145.01456，179.05215，251.06721，265.09452，269.11572，271.12561，277.09146，291.02561
22	senkyunolide S	7.77	C12H16O4	+CH3COO	240.09977	299.115	4.7	137.07473，221.05526
23	ligusticumside B	8.16	C23H30O12	+H	498.17373	499.1783	-5.4	135.08998、145.06858、163.08130
24	Salvilenone	8.81	C18H16O6	-H	292.14633	291.138	-3.6	275.10324，291.13483
25	ligusticumacid B	9.98	C12H14O3	+CH3COO	472.17333	531.1866	-1.1	133.07924，141.06961，151.05438，161.07552，311.09492，325.10097，449.12648
26	Acacetin-7-O-alpha-L-rhamno	10.25	C22H22O11	+CH3COO	430.12638	489.1362	-8.2	109.07905、161.06793、163.06821

	pyranoside							
27	propanetriol-a-arabinofuranosyl(1→4)	10.28	C20H34O17	+CH3COO	342.11621	401.1312	2.7	101.07673、113.07470
28	senkyunolide A	10.69	C12H16O2	-H	192.11503	191.106	-9.2	79.06561，107.06556，191.10589
29	cartorimine	10.91	C15H14O6	-H	302.07904	301.0739	7.1	117.09820，213.03289，123.02892，151.02892，235.03380
30	4,4-dimethylheptanoic acid	10.97	C7H14O2	+Cl	158.13068	193.0992	-4.6	139.15643、157.16574、193.13542
31	(E)-3-(3-hydroxy-4,5-dimethoxy-phenyl)acrylic acid	11.05	C11H12O5	+Na	224.06847	247.0589	5	355.10590，367.10547，379.10500，398.11318，167.07314，182.05518，290.10718，314.10095，355.10590，367.10547，369.12586，379.10501
32	L-2-Amino-3-(5-hydroxyindolyl)propionic acid	11.06	C11H12N2O3	-H	220.08479	219.0775	-0.2	93.08468，104.07650，120.07139，130.09257，132.07275，146.08668，158.08860，173.07545，202.07468，219.07738
33	Vitamin- G	11.07	C17H20N4O6	+HCOO	376.13828	421.1333	-7.6	71.06120，87.05867，221.05540
34	tetramethylpyrazine	11.24	C8H12N2	+CH3COO	136.10005	195.1155	8.4	135.09657、195.11555
35	chuanxiongdiolide R1	11.44	C47H80O18	-H	396.19367	395.1836	-7.1	73.07768，121.08096，129.06972
36	4-[(E)-4-(3,5-dimethoxy-4-oxo-1-cyclohexa-2,5-dienylidene)but-2-enylidene]-2,6-dimethoxycyclohexa-2,5-dien-1-one	12.63	C9H12O3	+HCOO	356.12599	401.1277	8.7	295.09755、321.07756、339.08564、353.15645
37	Pyrethrin II	15.77	C22H28O5	+CH3COO	372.19367	431.2053	-5.2	123.09627，135.09607，145.08093，147.09463，179.08378
38	ligusticumacid E	15.81	C12H14O3	+CH3COO	518.14243	577.1549	-2.4	121.08141，135.09607，145.08093，147.09463，159.09680
39	Kaempferide	16.11	C16H14O7	-H	300.06339	299.058	6.3	137.07678，165.03413，193.06332，227.07895，253.05788，

								257.08972，269.08867，271.06777
40	tinctorimine	16.76	C16H20N2O4	+HCOO	594.14589	639.1436	-0.8	257.08739，299.09519
41	2-methoxy-4-(3-methoxy-1-propenyl)-phenol	16.77	C11H14O3	+H	194.09429	195.1035	9.9	117.07449、149.09942
42	ligusticumside G	17.28	C12H16O3	+HCOO	368.11073	413.1105	3.8	133.08112，145.08160，149.07392，177.06435，293.07011，311.09109，455.11838
43	Luteolin-7-O-(6’’-O-acetyl)-β-glucopyranoside	17.75	C21H20O11	+CH3COO	490.11113	549.1275	4.6	133.08246，227.08134，229.09753，229.09753，229.09753，257.08670，283.06286，285.08068，549.12500
44	2-isopropyl-8-methylphenanthrene-3,4-dione	17.81	C18H16O2	+K	264.11503	303.08	6	155.08758、165.06118
45	(+)-Syringaresinol	17.82	C10H12O4	+HCOO	418.16277	463.1648	8.3	249.01119、265.19085
46	ethyl lithospermate	17.85	C16H22O6	+HCOO	502.16277	547.157	-7.3	123.09647，151.09082，199.08738，595.15316
47	salvianolic acid n	18.23	C30H38O14	+HCOO	494.1213	539.1205	1.9	95.06339，123.09530，297.07873，539.12087
48	quercetin_1	18.58	C15H10O7	-H	302.04265	301.0369	5.2	125.07565，135.09690，257.08715，301.02531
49	chuanxiongoside B	18.59	C25H32O12	+HCOO, +Cl	504.22068	549.2183	-1	311.09449，323.09130，325.10668，449.12421
50	paramiltioic acid	19.75	$C_{10}H_{16}O_4$	+Cl	332.16237	367.1334	4.4	329.16631，367.13291
51	danshenspiroketallactone	20.07	C20H30O5	+HCOO	282.12559	327.1219	-5.8	185.07409，197.09493，267.10647
52	astragalin_1	20.19	C21H20O11	+CH3COO	448.10056	507.117	5	117.08454、241.09552
53	isosalvianolic acid c	20.96	C10H10O5	+HCOO	492.10565	537.1066	5.1	109.08039，121.08240，123.09579，135.09611，149.07621，175.08846，179.08539，195.09448，197.09690，235.08874，263.07947，265.09353，293.08366，311.09599，323.03585，339.08567

54	1-β-ethylacrylate-7-aldehydo-β-carboline	21.4	C17H14N2O3	+Na	294.10044	317.0911	4.7	69.03404、115.09362、119.09056，157.10493，115.09362
55	phenyl(azido-1)-2-hydroxynaphthalene	21.41	C17H12O3	+Cl	248.09496	283.0653	3.4	107.06478，157.07754，212.09193，255.07434，325.10658
56	perlolyrine	22.8	C16H12N2O2	+Cl	264.08988	299.0598	1.8	179.08638，185.07422，299.05809
57	Isotanshinone I	23.19	C19H18O3	+HCOO	276.07864	321.0773	1.5	167.04906，275.09605，321.07741
58	Przewaquinone B	23.2	C20H24O6	+HCOO	292.07356	337.069	-8.2	235.08397，263.07975，337.06749
59	(6S)-6-hydroxy-1-methyl-6-methylol-8,9-dihydro-7H-naphtho[8,7-g]benzofuran-10,11-quinone	23.33	C19H18O5	-H	312.09977	311.092	-1.5	135.09659、161.07452、165.06956、207.09311
60	lirioresinol-A	23.37	$C_{15}H_{24}O_3$	+Cl	404.14712	439.113	-8.1	235.07104，237.10185，249.09047，263.08073，291.10962
61	cartormin	23.43	C27H29NO13	+H	575.16389	576.1703	-1.5	119.09004、191.03943
62	levistolide A	23.45	C24H28O4	+H	380.19876	381.2024	-9.6	89.07931、117.07440
63	safflow-yellow-A	23.46	C45H68O18	+Cl	594.15847	629.1323	7	161.07609，163.08071，191.03026，719.12560，721.13017
64	Danshenol B	24.6	$C_{11}H_{14}O_4$	+Cl	354.18311	389.1524	-0.3	219.09369，237.10091，239.12469，267.11024，269.12190，313.18281
65	aurantiamide acetate	25.08	C19H24N4O4	+CH3COO	444.20491	503.2216	5.6	146.06425、226.15254、250.15432、267.15672
66	3′,6,8′,3a-diligustilide	25.35	C24H26O6	-H	380.19876	379.1939	6.4	193.14861、209.16760、221.14415、247.17843
67	Heriguard	25.53	C13H10Cl2N4	+HCOO	354.09508	399.0906	-6.7	103.00006，131.01850，135.09636，173.07422，335.09131
68	ligusticumacid C	26.02	C15H20O4	+HCOO	516.16316	561.1571	-7.6	109.07995，123.05803，153.06960，167.04840，287.09627

69	Tilianin	26.23	C22H22O10	+CH3COO, +HCOO	446.1213	505.1361	1.9	255.06668，257.08900，285.08343，505.13780
70	dan-shexinkumc	26.39	C16H12O3	+H	252.07864	253.0861	0.7	261.09268、109.06974、143.08831、193.10297、221.09626、223.11202、233.09555、249.08927、251.10418
71	lithospermic acid	26.4	C27H22O12	+H	538.11113	539.1207	4.2	65.07442、107.08932、109.06974、111.08384、117.07420、123.08606、135.08428、137.06336、139.08030、163.08119、181.08922、295.09079、323.08279、411.09213、413.09829、447.12332、475.12196、493.12602、495.13556、521.11269、539.13077、663.11785、701.12919、719.13462
72	daidzein	26.41	C15H10O4	-H	254.05791	253.0532	10	93.08468，117.08589，135.05982，185.07473，197.07061，199.08720，221.05210，253.05788
73	Aethiopinone	26.84	C20H24O2	+Cl	296.17763	331.1478	2.3	171.18943、173.01456、185.06783、197.01567、229.16543、295.16832
74	6-o-syringyl-8-o-acetyl shanzhiside methyl ester_qt	26.96	C28H36O16	+Cl	466.14751	501.1125	-8.8	59.03856、73.03546、87.07684、89.07432、103.07854、355.16754、429.11356、447.17865
75	dihydrotanshinoneI	27.01	C18H14O3	+H	278.09429	279.0998	-6.4	221.09669、223.11538、251.11005、 277.08562、279.10011
76	miltirone II	28.63	C16H16O4	+Na	272.10486	295.0912	-9.6	93.07304、107.08920、223.11231、225.09298、239.10578、251.10512、255.10198
77	daidzin	28.88	C21H20O9	+CH3COO	416.11073	475.1292	9.7	93.08468，117.08589，211.05098，223.09048，251.07762，253.09414
78	(S,2E,4E)-6((2S,5R)-5-ethyltetrahydrofuran-2-yl)-6-hydroxy-4-methylhexa-2,4-dienoic acid	28.89	C13H20O4	+K	240.13616	279.0987	-2.3	123.08788，165.10816，221.10082
79	D-Phenylalanine	28.9	C9H11NO2	+CH3COO	165.07898	224.0941	5.9	147.09822、210.07712
80	banegasine	28.91	C9H11NO2	-H	204.08988	203.0838	5.8	129.04522、159.09532
81	kaempferol-3-O-β-D-glucopy	29.2	C21H18O12	+HCOO	610.15338	655.149	-3.9	283.09666，491.10084，593.14846

	ranosyl-7-O-β-D-glucopyranoside							
82	Monomethyl lithospermate	29.46	C28H24O12	-H	552.12678	551.1179	-2.9	123.09600，135.09680，179.08593，197.09347，249.09692，353.09847，449.12521，551.11783
83	dan-shexinkuma	30.19	C18H16O4	+H	296.10486	297.111	-4	251.10492、233.09410、223.11274
84	neocryptotanshinone ii	30.2	C19H22O3	-H	270.12559	269.1207	9	147.09822，185.07384，253.09211，267.10707，269.12218，295.10136，297.10812
85	boydenes A	30.21	C19H26O2	+HCOO	250.15689	295.1541	-3.5	233.11562、295.16267
86	folinic acid	30.41	C20H23N7O7	+CH3COO	473.1659	532.1843	8.6	382.15657，532.18384
87	6-hydroxykaempferol-6,7-di-O-beta-D-glucoside	30.85	C15H10O7	-H	626.1483	625.1417	1.1	117.05321、321.05436
88	Danshenol A	30.92	C21H20O4	+Cl	336.13616	371.1055	-0.3	219.09369，235.08397，249.09214，277.08787，291.10962
89	chuanxiongnolide L3	30.96	C24H32O4S	-H	340.13107	339.1222	-4.7	107.06472，108.07187，124.09595
90	oleic acid_2	31	C18H34O2	+HCOO	282.25588	327.2542	0.4	109.08067，327.25434
91	luteolin-7-0-B-D-glucoside	31.05	C21H20O11	+HCOO	580.14282	625.1439	4.6	109.08067、159.09389
92	palmitic acid_2	31.1	C16H32O2	+HCOO	256.24023	301.2397	4.2	59.05921，73.04099，87.05829，89.07524，101.07438，103.05601，131.08638
93	MEGxp0_000365	31.38	C34H37N3O6	+H	583.26824	584.2731	-4.2	147.08515、559.12780
94	angelicide	31.45	C24H28O4	+HCOO	382.21441	427.2152	6	267.11573、293.02641、295.11467
95	4,5-dehydro-diligustilide	31.99	C11H8BrN5	+HCOO	378.18311	423.1838	6	329.26581、331.27264
96	przewalskin	32.49	C18H24O2	+CH3COO	272.17763	331.1924	2.9	229.19119，255.11238，269.12430，331.18764
97	L-alpha-Palmitin	32.52	C51H98O6	-H	330.27701	329.2693	-1.2	137.14866，155.15894，157.17576，171.15339，183.18900，197.16507，199.18395，211.18246，225.10135，239.19255
98	zoomaric acid	32.57	C18H34O2	+HCOO	254.22458	299.224	3.9	125.14967，127.16335，163.16441，181.17422，209.16778

99	chuanxiongnolide L2	32.64	C12H14O3	+HCOO	412.22497	457.2231	-0.2	73.04108，121.08096，159.09624，161.07463
100	linoleic acid_1	32.71	C18H30O2	+HCOO	280.24023	325.2375	-2.8	321.12747，235.08688，325.24047
101	carnosol	32.75	C20H26O4	-H	330.18311	329.175	-2.6	123.05272，295.05272，297.02573，313.16372
102	methyl ester derived from angeolide	33.04	C9H10O2	+HCOO	412.22497	457.2227	-1	145.11728，287.24182
103	Daturic acid	33.25	C17H34O2	+HCOO	270.25588	315.2563	6.9	137.11332，165.10649
104	C09092	33.51		+HCOO	286.22967	331.2256	-6.9	267.10854、269.12825
105	Miltirone	33.58	C19H22O2	+HCOO	282.16198	327.158	-6.6	171.15203，173.07458，183.19016，211.18232，225.19926，327.15219
106	Ligla	33.62	C18H30O2	+HCOO	278.22458	323.2227	-0.3	153.04695，163.16519，233.20066，275.24483
107	5,6-dihydroxy-7-isopropyl-1,1-dimethyl-2,3-dihydrophenanthren-4-one	34.28	C19H22O3	-H	298.15689	297.151	4.8	121.04353、133.04635、149.05634、237.13422、239.15432、253.05554、253.14654、265.04666
108	Tanshilactone	34.35	C17H12O3	+H	264.07864	265.0867	2.8	137.09903、149.06759
109	Dehydromiltirone	34.54	C19H20O2	+HCOO	280.14633	325.1455	3.1	223.12199、325.14252
110	Satol	35.06	C18H36O	+HCOO	268.27662	313.2767	5.9	125.14859，125.14859，165.14132，313.27605
111	NERYLACETATE	37.68	C12H20O2	+K	196.14633	235.1113	7.9	177.10874、195.12323、233.13142
112	3-epicorosolic, acid	37.86	C30H48O4	+CH3COO	472.35526	531.3703	2.2	113.07565、155.10130、171.13171
113	chuanxiongoside A	39.11	C20H21NO10S	+HCOO	384.2148	429.2169	9.2	107.06472，163.05497，177.07045
