# Supplementary material for: Herbal Composition Inhibits Mitochondrial Oxidative Phosphorylation to Prevent HER2-Positive Breast Cancer and Identifies Potential Active Compounds
Source: Int J Mol Sci. 2025 Dec 12;26(24):11970. doi: 10.3390/ijms262411970 (PMC12733102; doi:10.3390/ijms262411970)
Supplement: Supplementary file 1 [file ijms-26-11970-s001.zip › Supplementary File S2.pdf]

Supplementary File S2

Table S2 Binding energies of active ingredient with target PDK1 protein

Number	Mol_id	Ingredient	Target protein scoring, total score
1	MO83	Monomethyl lithospermate	10.2779
2	MO8	icariside F2	9.7422
3	MO47	ethyl lithospermate	9.597
4	MO98	L-alpha-Palmitin	9.1847
5	MO111	Satol	8.9019
6	MO101	linoleic acid 1	8.2442
7	MO35	tetramethylpyrazine	8.1259
8	MO78	daidzin	8.0338
9	MO70	Tilianin	7.9444
10	MO27	Acacetin-7-O-alpha-L-rhamnopyranoside	7.6488
11	MO95	angelicide	7.5901
12	MO3	neocarthamin	7.5491
13	MO72	lithospermic acid	7.5286
14	MO114	chuanxiongoside A	7.3651
15	MO53	astragalin1	7.3581
16	MO99	zoomaric acid	7.1071
17	MO104	Daturic acid	7.0096
18	MO112	NERYLACETATE	6.7276
19	MO54	isosalvianolic acid c	6.7243
20	MO87	folinic acid	6.6992
21	MO61	lirioresinol-A	6.6583
22	MO48	salvianolic acid n	6.3104
23	MO7	Naringin	6.2303
24	MO23	senkyunolide S	6.1509
25	MO33	L-2-Amino-3-(5-hydroxyindolyl)propionic acid	6.0648
26	MO25	Salvilenone	6.0622

27	MO42	2-methoxy-4-(3-methoxy-1-propenyl)-phenol	5.9395
28	MO59	Przewaquinone B	5.8703
29	MO79	(S,2E,4E)-6((2S,5R)-5-ethyltetrahydrofuran-2-yl)-6-hydroxy-4-methylhexa-2,4-dienoic acid	5.7444
30	MO10	hydroxysafflor yellow B	5.6332
31	MO40	Kaempferide	5.5518
32	MO66	aurantiamide acetate	5.5025
33	MO68	Heriguard	5.4596
34	MO85	neocryptotanshinone ii	5.4382
35	MO74	Aethiopinone	5.4001
36	MO105	C09092	5.3457
37	MO46	(+)-Syringaresinol	5.2403
38	MO106	Miltirone	5.2095
39	MO30	cartorimine	5.1452
40	MO81	banegasine	5.1036
41	MO102	carnosol	5.0899
42	MO31	4,4-dimethylheptanoic acid	5.0108
43	MO57	perlolyrine	5.0098
